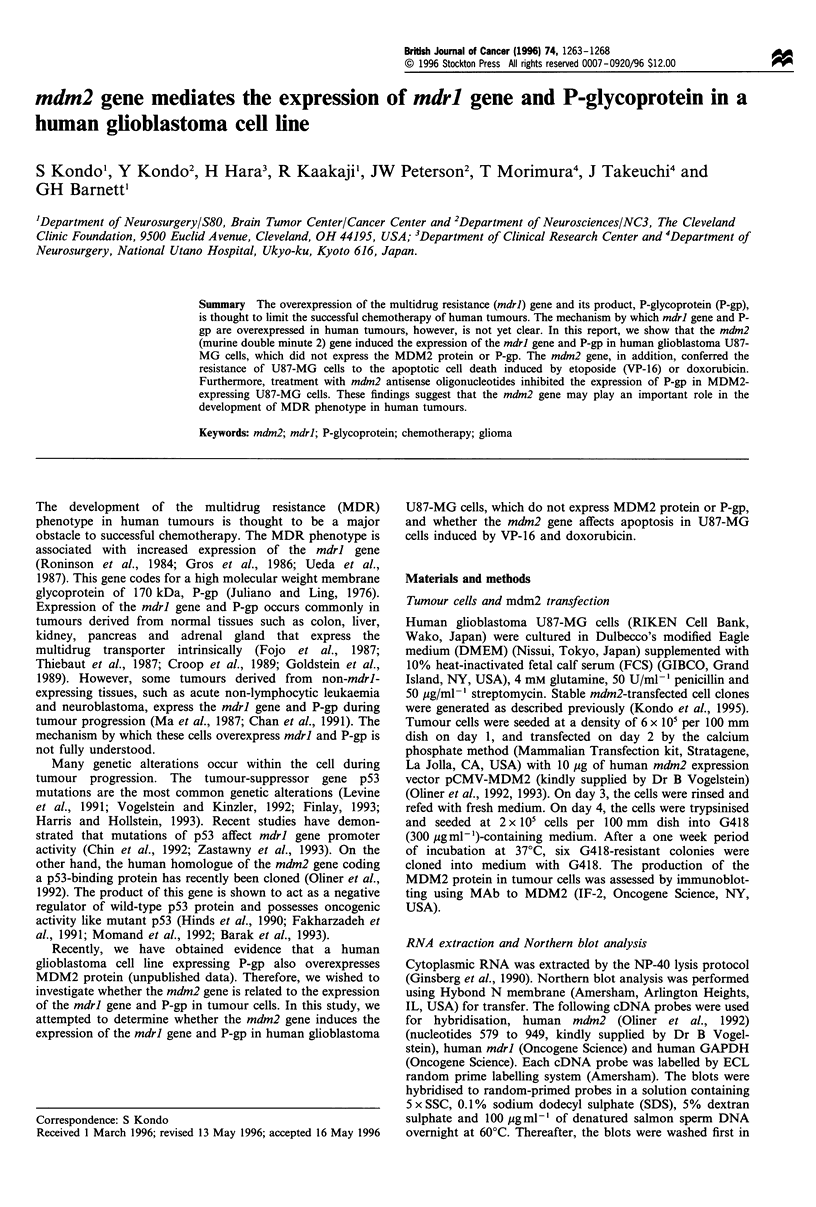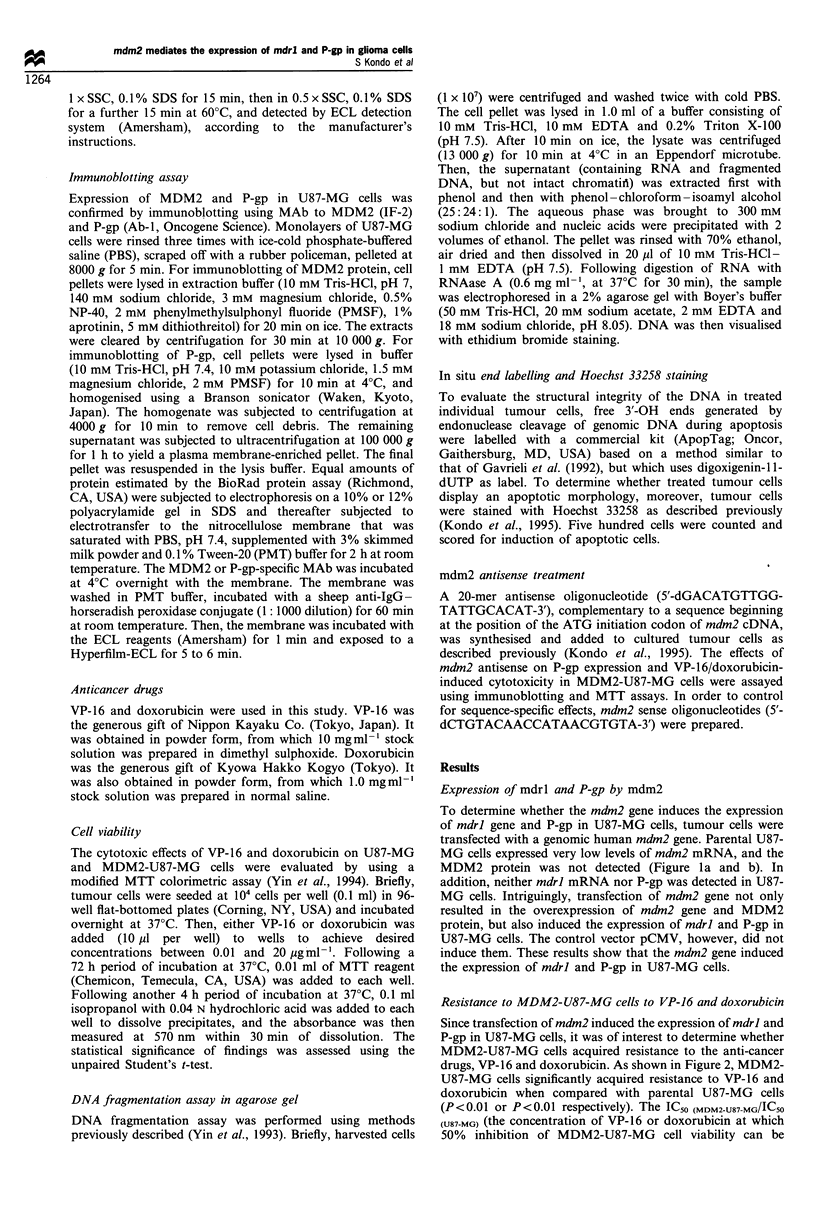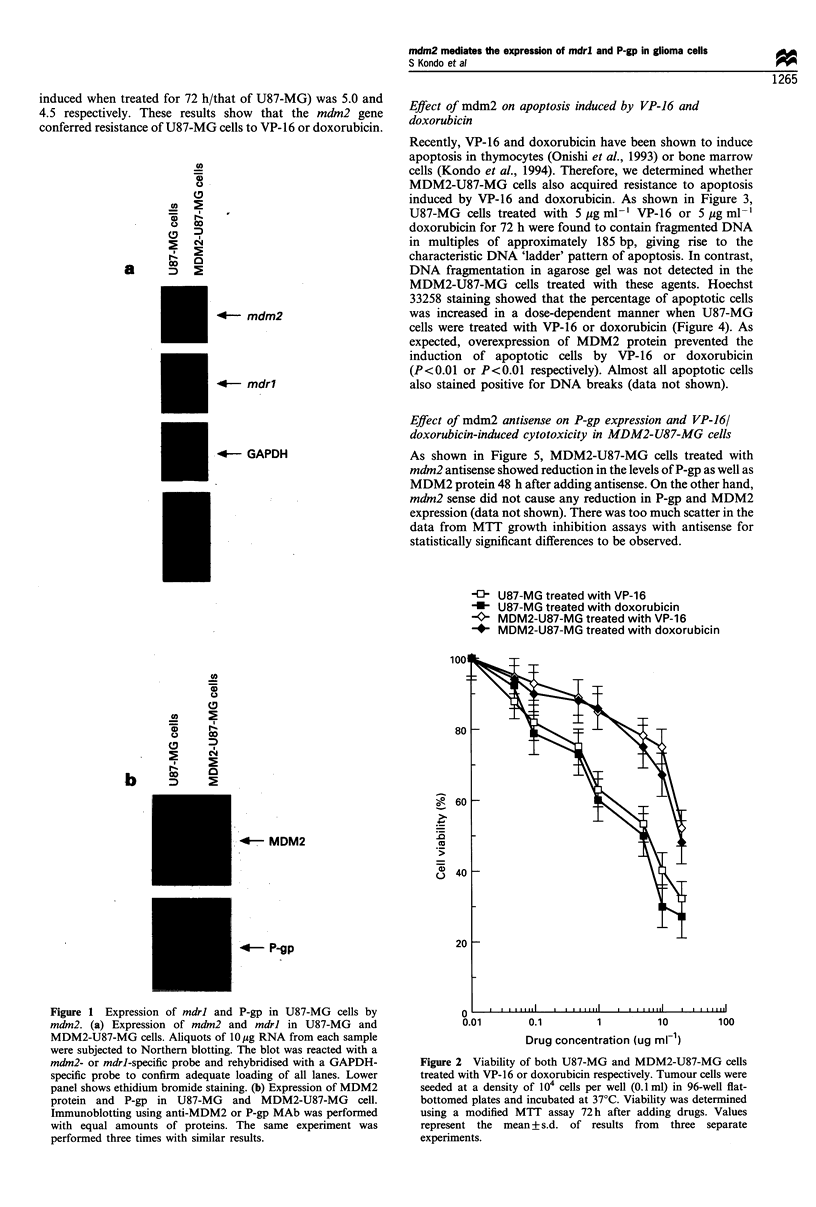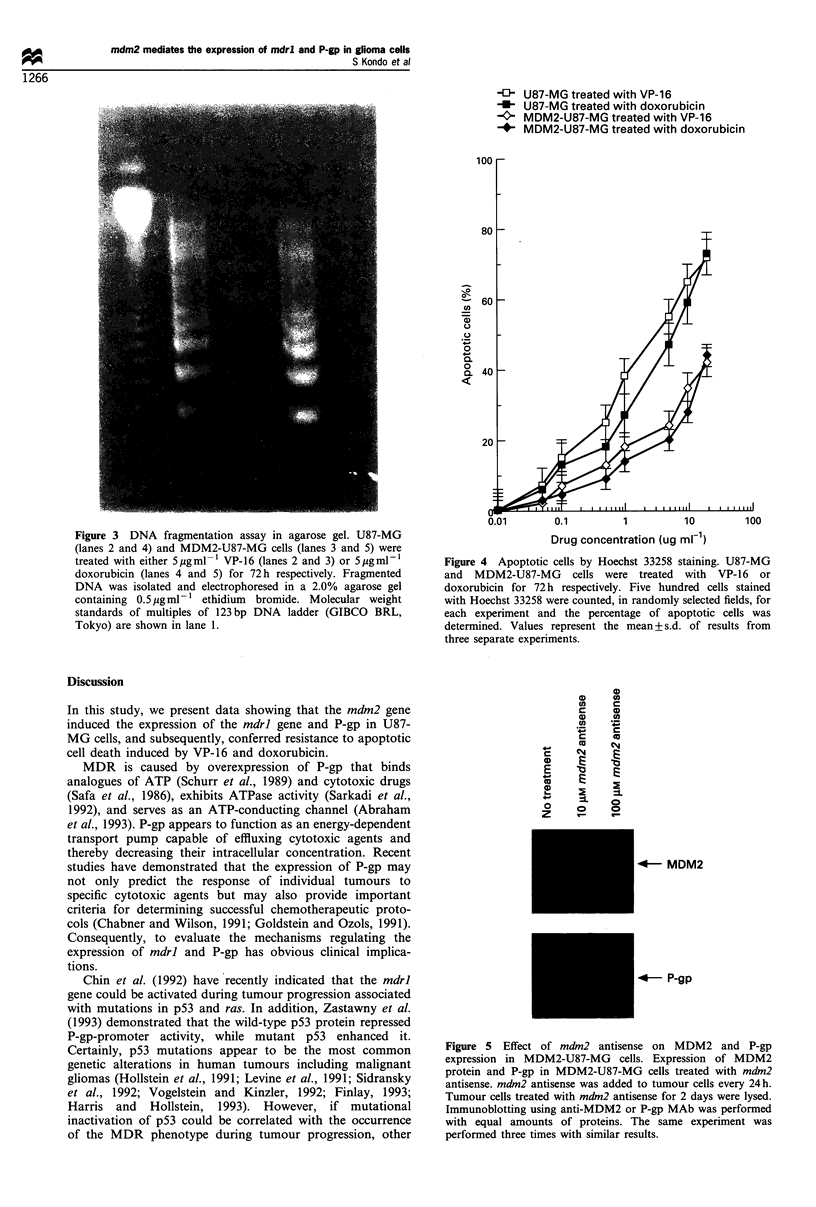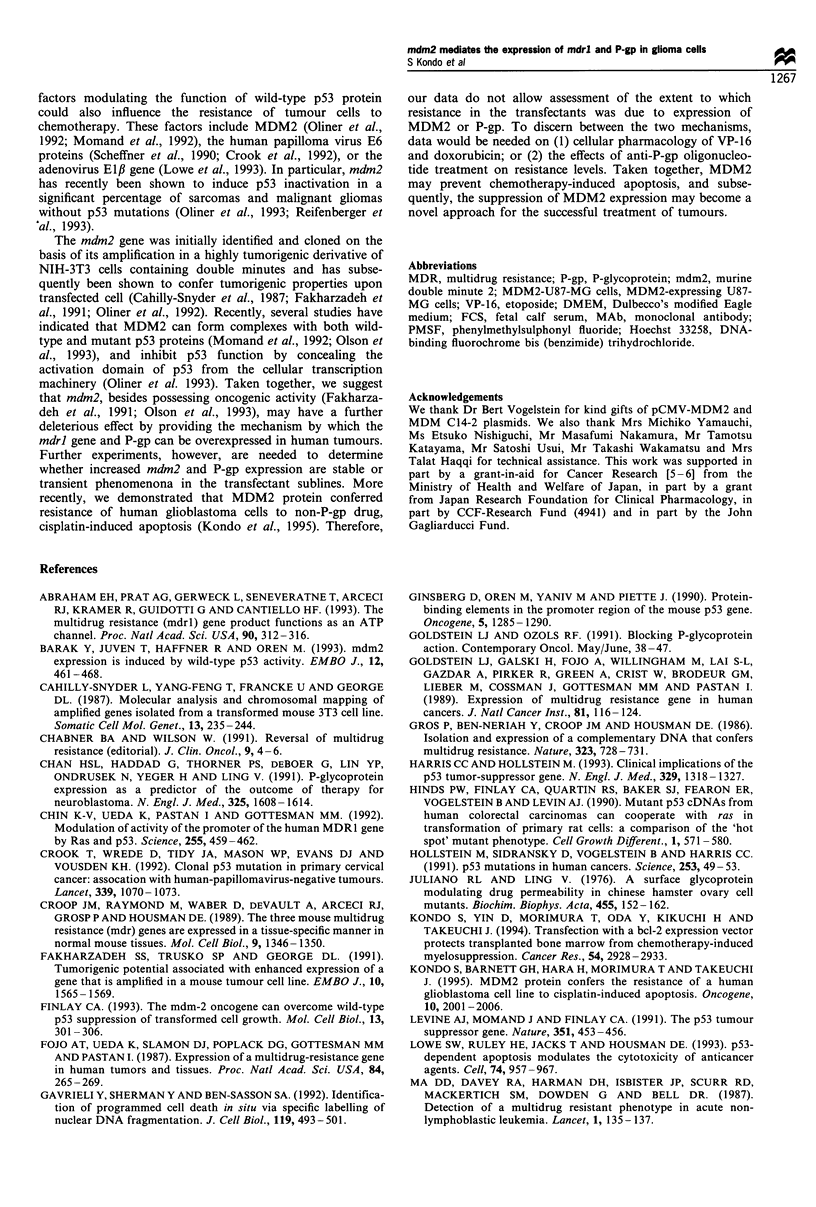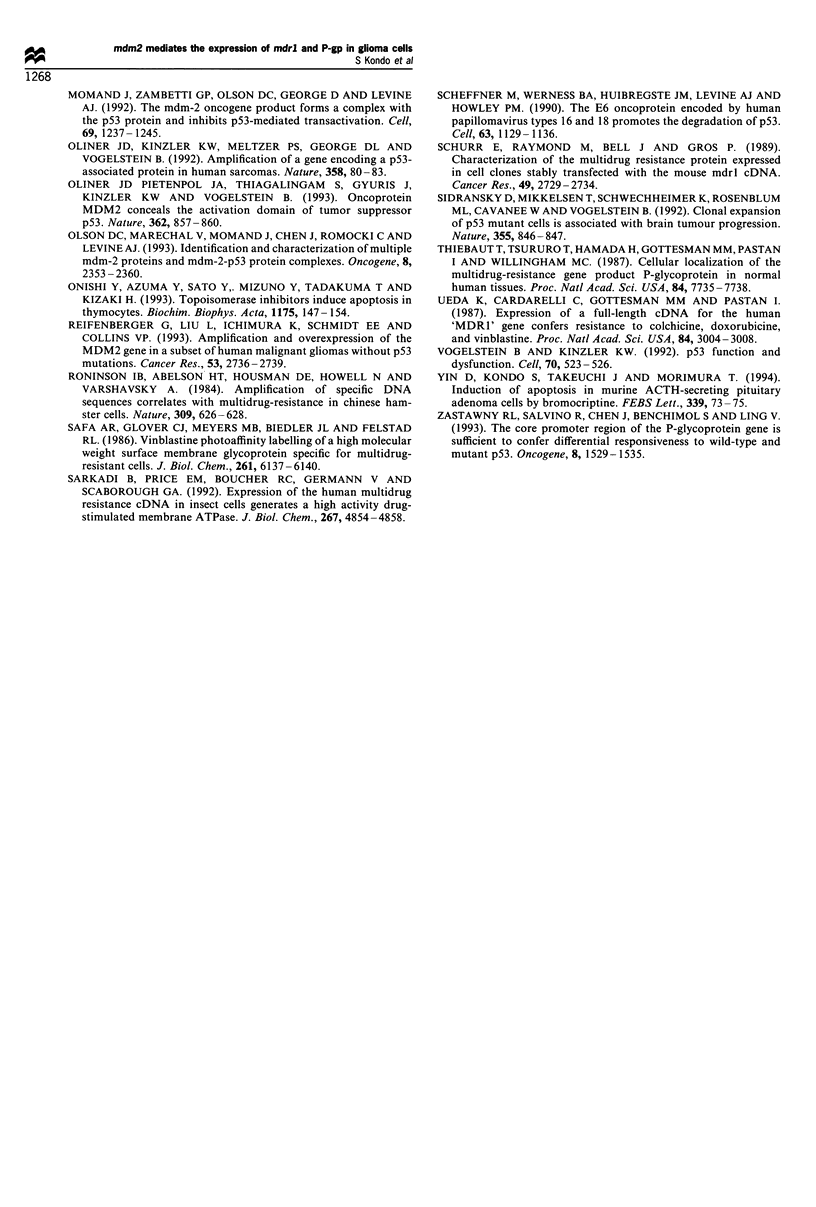# mdm2 gene mediates the expression of mdr1 gene and P-glycoprotein in a human glioblastoma cell line.

**DOI:** 10.1038/bjc.1996.527

**Published:** 1996-10

**Authors:** S. Kondo, Y. Kondo, H. Hara, R. Kaakaji, J. W. Peterson, T. Morimura, J. Takeuchi, G. H. Barnett

**Affiliations:** Department of Neurosurgery/S80, Cleveland Clinic Foundation, OH 44195, USA.

## Abstract

**Images:**


British Journal of Cancer (1996) 74, 1263-1268
© 1996 Stockton Press All rights reserved 0007-0920/96 $12.00

### *mdm2* gene mediates the expression of *mdr1* gene and P-glycoprotein in a human glioblastoma cell line

S Kondo[1], Y Kondo[2], H Hara[3], R Kaakaji[1], JW Peterson[2], T Morimura[4], J Takeuchi[4] and GH Barnett[1]

[1]*Department of Neurosurgery/S80, Brain Tumor Center/Cancer Center and* [2]*Department of Neurosciences/NC3, The Cleveland Clinic Foundation, 9500 Euclid Avenue, Cleveland, OH 44195, USA;* [3]*Department of Clinical Research Center and* [4]*Department of Neurosurgery, National Utano Hospital, Ukyo-ku, Kyoto 616, Japan.*

**Summary** The overexpression of the multidrug resistance (*mdr1*) gene and its product, P-glycoprotein (P-gp), is thought to limit the successful chemotherapy of human tumours. The mechanism by which *mdr1* gene and P-gp are overexpressed in human tumours, however, is not yet clear. In this report, we show that the *mdm2* (murine double minute 2) gene induced the expression of the *mdr1* gene and P-gp in human glioblastoma U87-MG cells, which did not express the MDM2 protein or P-gp. The *mdm2* gene, in addition, conferred the resistance of U87-MG cells to the apoptotic cell death induced by etoposide (VP-16) or doxorubicin. Furthermore, treatment with *mdm2* antisense oligonucleotides inhibited the expression of P-gp in MDM2-expressing U87-MG cells. These findings suggest that the *mdm2* gene may play an important role in the development of MDR phenotype in human tumours.

**Keywords:** *mdm2*; *mdr1*; P-glycoprotein; chemotherapy; glioma

The development of the multidrug resistance (MDR) phenotype in human tumours is thought to be a major obstacle to successful chemotherapy. The MDR phenotype is associated with increased expression of the *mdr1* gene (Roninson *et al.*, 1984; Gros *et al.*, 1986; Ueda *et al.*, 1987). This gene codes for a high molecular weight membrane glycoprotein of 170 kDa, P-gp (Juliano and Ling, 1976). Expression of the *mdr1* gene and P-gp occurs commonly in tumours derived from normal tissues such as colon, liver, kidney, pancreas and adrenal gland that express the multidrug transporter intrinsically (Fojo *et al.*, 1987; Thiebaut *et al.*, 1987; Croop *et al.*, 1989; Goldstein *et al.*, 1989). However, some tumours derived from non-*mdr1*-expressing tissues, such as acute non-lymphocytic leukaemia and neuroblastoma, express the *mdr1* gene and P-gp during tumour progression (Ma *et al.*, 1987; Chan *et al.*, 1991). The mechanism by which these cells overexpress *mdr1* and P-gp is not fully understood.

Many genetic alterations occur within the cell during tumour progression. The tumour-suppressor gene p53 mutations are the most common genetic alterations (Levine *et al.*, 1991; Vogelstein and Kinzler, 1992; Finlay, 1993; Harris and Hollstein, 1993). Recent studies have demonstrated that mutations of p53 affect *mdr1* gene promoter activity (Chin *et al.*, 1992; Zastawny *et al.*, 1993). On the other hand, the human homologue of the *mdm2* gene coding a p53-binding protein has recently been cloned (Oliner *et al.*, 1992). The product of this gene is shown to act as a negative regulator of wild-type p53 protein and possesses oncogenic activity like mutant p53 (Hinds *et al.*, 1990; Fakharzadeh *et al.*, 1991; Momand *et al.*, 1992; Barak *et al.*, 1993).

Recently, we have obtained evidence that a human glioblastoma cell line expressing P-gp also overexpresses MDM2 protein (unpublished data). Therefore, we wished to investigate whether the *mdm2* gene is related to the expression of the *mdr1* gene and P-gp in tumour cells. In this study, we attempted to determine whether the *mdm2* gene induces the expression of the *mdr1* gene and P-gp in human glioblastoma U87-MG cells, which do not express MDM2 protein or P-gp, and whether the *mdm2* gene affects apoptosis in U87-MG cells induced by VP-16 and doxorubicin.

Correspondence: S Kondo
Received 1 March 1996; revised 13 May 1996; accepted 16 May 1996

### Materials and methods

### *Tumour cells and* mdm2 *transfection*

Human glioblastoma U87-MG cells (RIKEN Cell Bank, Wako, Japan) were cultured in Dulbecco's modified Eagle medium (DMEM) (Nissui, Tokyo, Japan) supplemented with 10% heat-inactivated fetal calf serum (FCS) (GIBCO, Grand Island, NY, USA), 4 mM glutamine, 50 U/ml$^{-1}$ penicillin and 50 $\mu$g/ml$^{-1}$ streptomycin. Stable *mdm2*-transfected cell clones were generated as described previously (Kondo *et al.*, 1995). Tumour cells were seeded at a density of $6 \times 10^5$ per 100 mm dish on day 1, and transfected on day 2 by the calcium phosphate method (Mammalian Transfection kit, Stratagene, La Jolla, CA, USA) with 10 $\mu$g of human *mdm2* expression vector pCMV-MDM2 (kindly supplied by Dr B Vogelstein) (Oliner *et al.*, 1992, 1993). On day 3, the cells were rinsed and refed with fresh medium. On day 4, the cells were trypsinised and seeded at $2 \times 10^5$ cells per 100 mm dish into G418 (300 $\mu$g ml$^{-1}$)-containing medium. After a one week period of incubation at 37°C, six G418-resistant colonies were cloned into medium with G418. The production of the MDM2 protein in tumour cells was assessed by immunoblotting using MAb to MDM2 (IF-2, Oncogene Science, NY, USA).

### *RNA extraction and Northern blot analysis*

Cytoplasmic RNA was extracted by the NP-40 lysis protocol (Ginsberg *et al.*, 1990). Northern blot analysis was performed using Hybond N membrane (Amersham, Arlington Heights, IL, USA) for transfer. The following cDNA probes were used for hybridisation, human *mdm2* (Oliner *et al.*, 1992) (nucleotides 579 to 949, kindly supplied by Dr B Vogelstein), human *mdr1* (Oncogene Science) and human GAPDH (Oncogene Science). Each cDNA probe was labelled by ECL random prime labelling system (Amersham). The blots were hybridised to random-primed probes in a solution containing $5 \times$SSC, 0.1% sodium dodecyl sulphate (SDS), 5% dextran sulphate and 100 $\mu$g ml$^{-1}$ of denatured salmon sperm DNA overnight at 60°C. Thereafter, the blots were washed first in

1 × SSC, 0.1% SDS for 15 min, then in 0.5 × SSC, 0.1% SDS for a further 15 min at 60°C, and detected by ECL detection system (Amersham), according to the manufacturer's instructions.

*Immunoblotting assay*

Expression of MDM2 and P-gp in U87-MG cells was confirmed by immunoblotting using MAb to MDM2 (IF-2) and P-gp (Ab-1, Oncogene Science). Monolayers of U87-MG cells were rinsed three times with ice-cold phosphate-buffered saline (PBS), scraped off with a rubber policeman, pelleted at 8000 ***g*** for 5 min. For immunoblotting of MDM2 protein, cell pellets were lysed in extraction buffer (10 mM Tris-HCl, pH 7, 140 mM sodium chloride, 3 mM magnesium chloride, 0.5% NP-40, 2 mM phenylmethylsulphonyl fluoride (PMSF), 1% aprotinin, 5 mM dithiothreitol) for 20 min on ice. The extracts were cleared by centrifugation for 30 min at 10 000 ***g***. For immunoblotting of P-gp, cell pellets were lysed in buffer (10 mM Tris-HCl, pH 7.4, 10 mM potassium chloride, 1.5 mM magnesium chloride, 2 mM PMSF) for 10 min at 4°C, and homogenised using a Branson sonicator (Waken, Kyoto, Japan). The homogenate was subjected to centrifugation at 4000 ***g*** for 10 min to remove cell debris. The remaining supernatant was subjected to ultracentrifugation at 100 000 ***g*** for 1 h to yield a plasma membrane-enriched pellet. The final pellet was resuspended in the lysis buffer. Equal amounts of protein estimated by the BioRad protein assay (Richmond, CA, USA) were subjected to electrophoresis on a 10% or 12% polyacrylamide gel in SDS and thereafter subjected to electrotransfer to the nitrocellulose membrane that was saturated with PBS, pH 7.4, supplemented with 3% skimmed milk powder and 0.1% Tween-20 (PMT) buffer for 2 h at room temperature. The MDM2 or P-gp-specific MAb was incubated at 4°C overnight with the membrane. The membrane was washed in PMT buffer, incubated with a sheep anti-IgG–horseradish peroxidase conjugate (1 : 1000 dilution) for 60 min at room temperature. Then, the membrane was incubated with the ECL reagents (Amersham) for 1 min and exposed to a Hyperfilm-ECL for 5 to 6 min.

*Anticancer drugs*

VP-16 and doxorubicin were used in this study. VP-16 was the generous gift of Nippon Kayaku Co. (Tokyo, Japan). It was obtained in powder form, from which 10 $mg\,ml^{-1}$ stock solution was prepared in dimethyl sulphoxide. Doxorubicin was the generous gift of Kyowa Hakko Kogyo (Tokyo). It was also obtained in powder form, from which 1.0 $mg\,ml^{-1}$ stock solution was prepared in normal saline.

*Cell viability*

The cytotoxic effects of VP-16 and doxorubicin on U87-MG and MDM2-U87-MG cells were evaluated by using a modified MTT colorimetric assay (Yin *et al.*, 1994). Briefly, tumour cells were seeded at $10^4$ cells per well (0.1 ml) in 96-well flat-bottomed plates (Corning, NY, USA) and incubated overnight at 37°C. Then, either VP-16 or doxorubicin was added (10 $\mu l$ per well) to wells to achieve desired concentrations between 0.01 and 20 $\mu g\,ml^{-1}$. Following a 72 h period of incubation at 37°C, 0.01 ml of MTT reagent (Chemicon, Temecula, CA, USA) was added to each well. Following another 4 h period of incubation at 37°C, 0.1 ml isopropanol with 0.04 N hydrochloric acid was added to each well to dissolve precipitates, and the absorbance was then measured at 570 nm within 30 min of dissolution. The statistical significance of findings was assessed using the unpaired Student's *t*-test.

*DNA fragmentation assay in agarose gel*

DNA fragmentation assay was performed using methods previously described (Yin *et al.*, 1993). Briefly, harvested cells ($1 \times 10^7$) were centrifuged and washed twice with cold PBS. The cell pellet was lysed in 1.0 ml of a buffer consisting of 10 mM Tris-HCl, 10 mM EDTA and 0.2% Triton X-100 (pH 7.5). After 10 min on ice, the lysate was centrifuged (13 000 ***g***) for 10 min at 4°C in an Eppendorf microtube. Then, the supernatant (containing RNA and fragmented DNA, but not intact chromatin) was extracted first with phenol and then with phenol–chloroform–isoamyl alcohol (25 : 24 : 1). The aqueous phase was brought to 300 mM sodium chloride and nucleic acids were precipitated with 2 volumes of ethanol. The pellet was rinsed with 70% ethanol, air dried and then dissolved in 20 $\mu l$ of 10 mM Tris-HCl–1 mM EDTA (pH 7.5). Following digestion of RNA with RNAase A (0.6 $mg\,ml^{-1}$, at 37°C for 30 min), the sample was electrophoresed in a 2% agarose gel with Boyer's buffer (50 mM Tris-HCl, 20 mM sodium acetate, 2 mM EDTA and 18 mM sodium chloride, pH 8.05). DNA was then visualised with ethidium bromide staining.

In situ *end labelling and Hoechst 33258 staining*

To evaluate the structural integrity of the DNA in treated individual tumour cells, free 3′-OH ends generated by endonuclease cleavage of genomic DNA during apoptosis were labelled with a commercial kit (ApopTag; Oncor, Gaithersburg, MD, USA) based on a method similar to that of Gavrieli *et al.* (1992), but which uses digoxigenin-11-dUTP as label. To determine whether treated tumour cells display an apoptotic morphology, moreover, tumour cells were stained with Hoechst 33258 as described previously (Kondo *et al.*, 1995). Five hundred cells were counted and scored for induction of apoptotic cells.

mdm2 *antisense treatment*

A 20-mer antisense oligonucleotide (5′-dGACATGTTGG-TATTGCACAT-3′), complementary to a sequence beginning at the position of the ATG initiation codon of *mdm2* cDNA, was synthesised and added to cultured tumour cells as described previously (Kondo *et al.*, 1995). The effects of *mdm2* antisense on P-gp expression and VP-16/doxorubicin-induced cytotoxicity in MDM2-U87-MG cells were assayed using immunoblotting and MTT assays. In order to control for sequence-specific effects, *mdm2* sense oligonucleotides (5′-dCTGTACAACCATAACGTGTA-3′) were prepared.

### Results

*Expression of* mdr1 *and P-gp by* mdm2

To determine whether the *mdm2* gene induces the expression of *mdr1* gene and P-gp in U87-MG cells, tumour cells were transfected with a genomic human *mdm2* gene. Parental U87-MG cells expressed very low levels of *mdm2* mRNA, and the MDM2 protein was not detected (Figure 1a and b). In addition, neither *mdr1* mRNA nor P-gp was detected in U87-MG cells. Intriguingly, transfection of *mdm2* gene not only resulted in the overexpression of *mdm2* gene and MDM2 protein, but also induced the expression of *mdr1* and P-gp in U87-MG cells. The control vector pCMV, however, did not induce them. These results show that the *mdm2* gene induced the expression of *mdr1* and P-gp in U87-MG cells.

*Resistance to MDM2-U87-MG cells to VP-16 and doxorubicin*

Since transfection of *mdm2* induced the expression of *mdr1* and P-gp in U87-MG cells, it was of interest to determine whether MDM2-U87-MG cells acquired resistance to the anti-cancer drugs, VP-16 and doxorubicin. As shown in Figure 2, MDM2-U87-MG cells significantly acquired resistance to VP-16 and doxorubicin when compared with parental U87-MG cells ($P < 0.01$ or $P < 0.01$ respectively). The $IC_{50\ (MDM2\text{-}U87\text{-}MG)}/IC_{50\ (U87\text{-}MG)}$ (the concentration of VP-16 or doxorubicin at which 50% inhibition of MDM2-U87-MG cell viability can be induced when treated for 72 h/that of U87-MG) was 5.0 and 4.5 respectively. These results show that the *mdm2* gene conferred resistance of U87-MG cells to VP-16 or doxorubicin.

**Figure 1** Expression of *mdr1* and P-gp in U87-MG cells by *mdm2*. (**a**) Expression of *mdm2* and *mdr1* in U87-MG and MDM2-U87-MG cells. Aliquots of 10 μg RNA from each sample were subjected to Northern blotting. The blot was reacted with a *mdm2*- or *mdr1*-specific probe and rehybridised with a GAPDH-specific probe to confirm adequate loading of all lanes. Lower panel shows ethidium bromide staining. (**b**) Expression of MDM2 protein and P-gp in U87-MG and MDM2-U87-MG cell. Immunoblotting using anti-MDM2 or P-gp MAb was performed with equal amounts of proteins. The same experiment was performed three times with similar results.

*Effect of* mdm2 *on apoptosis induced by VP-16 and doxorubicin*

Recently, VP-16 and doxorubicin have been shown to induce apoptosis in thymocytes (Onishi *et al.*, 1993) or bone marrow cells (Kondo *et al.*, 1994). Therefore, we determined whether MDM2-U87-MG cells also acquired resistance to apoptosis induced by VP-16 and doxorubicin. As shown in Figure 3, U87-MG cells treated with 5 μg $ml^{-1}$ VP-16 or 5 μg $ml^{-1}$ doxorubicin for 72 h were found to contain fragmented DNA in multiples of approximately 185 bp, giving rise to the characteristic DNA 'ladder' pattern of apoptosis. In contrast, DNA fragmentation in agarose gel was not detected in the MDM2-U87-MG cells treated with these agents. Hoechst 33258 staining showed that the percentage of apoptotic cells was increased in a dose-dependent manner when U87-MG cells were treated with VP-16 or doxorubicin (Figure 4). As expected, overexpression of MDM2 protein prevented the induction of apoptotic cells by VP-16 or doxorubicin ($P<0.01$ or $P<0.01$ respectively). Almost all apoptotic cells also stained positive for DNA breaks (data not shown).

*Effect of* mdm2 *antisense on P-gp expression and VP-16/doxorubicin-induced cytotoxicity in MDM2-U87-MG cells*

As shown in Figure 5, MDM2-U87-MG cells treated with *mdm2* antisense showed reduction in the levels of P-gp as well as MDM2 protein 48 h after adding antisense. On the other hand, *mdm2* sense did not cause any reduction in P-gp and MDM2 expression (data not shown). There was too much scatter in the data from MTT growth inhibition assays with antisense for statistically significant differences to be observed.

**Figure 2** Viability of both U87-MG and MDM2-U87-MG cells treated with VP-16 or doxorubicin respectively. Tumour cells were seeded at a density of $10^4$ cells per well (0.1 ml) in 96-well flat-bottomed plates and incubated at 37°C. Viability was determined using a modified MTT assay 72 h after adding drugs. Values represent the mean±s.d. of results from three separate experiments.

**Figure 3** DNA fragmentation assay in agarose gel. U87-MG (lanes 2 and 4) and MDM2-U87-MG cells (lanes 3 and 5) were treated with either 5 $\mu$g ml$^{-1}$ VP-16 (lanes 2 and 3) or 5 $\mu$g ml$^{-1}$ doxorubicin (lanes 4 and 5) for 72 h respectively. Fragmented DNA was isolated and electrophoresed in a 2.0% agarose gel containing 0.5 $\mu$g ml$^{-1}$ ethidium bromide. Molecular weight standards of multiples of 123 bp DNA ladder (GIBCO BRL, Tokyo) are shown in lane 1.

**Figure 4** Apoptotic cells by Hoechst 33258 staining. U87-MG and MDM2-U87-MG cells were treated with VP-16 or doxorubicin for 72 h respectively. Five hundred cells stained with Hoechst 33258 were counted, in randomly selected fields, for each experiment and the percentage of apoptotic cells was determined. Values represent the mean $\pm$ s.d. of results from three separate experiments.

### Discussion

In this study, we present data showing that the *mdm2* gene induced the expression of the *mdr1* gene and P-gp in U87-MG cells, and subsequently, conferred resistance to apoptotic cell death induced by VP-16 and doxorubicin.

MDR is caused by overexpression of P-gp that binds analogues of ATP (Schurr *et al*., 1989) and cytotoxic drugs (Safa *et al*., 1986), exhibits ATPase activity (Sarkadi *et al*., 1992), and serves as an ATP-conducting channel (Abraham *et al*., 1993). P-gp appears to function as an energy-dependent transport pump capable of effluxing cytotoxic agents and thereby decreasing their intracellular concentration. Recent studies have demonstrated that the expression of P-gp may not only predict the response of individual tumours to specific cytotoxic agents but may also provide important criteria for determining successful chemotherapeutic protocols (Chabner and Wilson, 1991; Goldstein and Ozols, 1991). Consequently, to evaluate the mechanisms regulating the expression of *mdr1* and P-gp has obvious clinical implications.

Chin *et al*. (1992) have recently indicated that the *mdr1* gene could be activated during tumour progression associated with mutations in p53 and *ras*. In addition, Zastawny *et al*. (1993) demonstrated that the wild-type p53 protein repressed P-gp-promoter activity, while mutant p53 enhanced it. Certainly, p53 mutations appear to be the most common genetic alterations in human tumours including malignant gliomas (Hollstein *et al*., 1991; Levine *et al*., 1991; Sidransky *et al*., 1992; Vogelstein and Kinzler, 1992; Finlay, 1993; Harris and Hollstein, 1993). However, if mutational inactivation of p53 could be correlated with the occurrence of the MDR phenotype during tumour progression, other

**Figure 5** Effect of *mdm2* antisense on MDM2 and P-gp expression in MDM2-U87-MG cells. Expression of MDM2 protein and P-gp in MDM2-U87-MG cells treated with *mdm2* antisense. *mdm2* antisense was added to tumour cells every 24 h. Tumour cells treated with *mdm2* antisense for 2 days were lysed. Immunoblotting using anti-MDM2 or P-gp MAb was performed with equal amounts of proteins. The same experiment was performed three times with similar results.

factors modulating the function of wild-type p53 protein could also influence the resistance of tumour cells to chemotherapy. These factors include MDM2 (Oliner *et al*., 1992; Momand *et al*., 1992), the human papilloma virus E6 proteins (Scheffner *et al*., 1990; Crook *et al*., 1992), or the adenovirus E1$\beta$ gene (Lowe *et al*., 1993). In particular, *mdm2* has recently been shown to induce p53 inactivation in a significant percentage of sarcomas and malignant gliomas without p53 mutations (Oliner *et al*., 1993; Reifenberger *et al*., 1993).

The *mdm2* gene was initially identified and cloned on the basis of its amplification in a highly tumorigenic derivative of NIH-3T3 cells containing double minutes and has subsequently been shown to confer tumorigenic properties upon transfected cell (Cahilly-Snyder *et al*., 1987; Fakharzadeh *et al*., 1991; Oliner *et al*., 1992). Recently, several studies have indicated that MDM2 can form complexes with both wild-type and mutant p53 proteins (Momand *et al*., 1992; Olson *et al*., 1993), and inhibit p53 function by concealing the activation domain of p53 from the cellular transcription machinery (Oliner *et al*. 1993). Taken together, we suggest that *mdm2*, besides possessing oncogenic activity (Fakharzadeh *et al*., 1991; Olson *et al*., 1993), may have a further deleterious effect by providing the mechanism by which the *mdr1* gene and P-gp can be overexpressed in human tumours. Further experiments, however, are needed to determine whether increased *mdm2* and P-gp expression are stable or transient phenomenona in the transfectant sublines. More recently, we demonstrated that MDM2 protein conferred resistance of human glioblastoma cells to non-P-gp drug, cisplatin-induced apoptosis (Kondo *et al*., 1995). Therefore, our data do not allow assessment of the extent to which resistance in the transfectants was due to expression of MDM2 or P-gp. To discern between the two mechanisms, data would be needed on (1) cellular pharmacology of VP-16 and doxorubicin; or (2) the effects of anti-P-gp oligonucleotide treatment on resistance levels. Taken together, MDM2 may prevent chemotherapy-induced apoptosis, and subsequently, the suppression of MDM2 expression may become a novel approach for the successful treatment of tumours.

### Abbreviations

MDR, multidrug resistance; P-gp, P-glycoprotein; mdm2, murine double minute 2; MDM2-U87-MG cells, MDM2-expressing U87-MG cells; VP-16, etoposide; DMEM, Dulbecco's modified Eagle medium; FCS, fetal calf serum, MAb, monoclonal antibody; PMSF, phenylmethylsulphonyl fluoride; Hoechst 33258, DNA-binding fluorochrome bis (benzimide) trihydrochloride.

### Acknowledgements

We thank Dr Bert Vogelstein for kind gifts of pCMV-MDM2 and MDM C14-2 plasmids. We also thank Mrs Michiko Yamauchi, Ms Etsuko Nishiguchi, Mr Masafumi Nakamura, Mr Tamotsu Katayama, Mr Satoshi Usui, Mr Takashi Wakamatsu and Mrs Talat Haqqi for technical assistance. This work was supported in part by a grant-in-aid for Cancer Research [5–6] from the Ministry of Health and Welfare of Japan, in part by a grant from Japan Research Foundation for Clinical Pharmacology, in part by CCF-Research Fund (4941) and in part by the John Gagliarducci Fund.

### References

ABRAHAM EH, PRAT AG, GERWECK L, SENEVERATNE T, ARCECI RJ, KRAMER R, GUIDOTTI G AND CANTIELLO HF. (1993). The multidrug resistance (mdr1) gene product functions as an ATP channel. *Proc. Natl Acad. Sci. USA*, **90**, 312–316.

BARAK Y, JUVEN T, HAFFNER R AND OREN M. (1993). mdm2 expression is induced by wild-type p53 activity. *EMBO J*., **12**, 461–468.

CAHILLY-SNYDER L, YANG-FENG T, FRANCKE U AND GEORGE DL. (1987). Molecular analysis and chromosomal mapping of amplified genes isolated from a transformed mouse 3T3 cell line. *Somatic Cell Mol. Genet*., **13**, 235–244.

CHABNER BA AND WILSON W. (1991). Reversal of multidrug resistance (editorial). *J. Clin. Oncol*., **9**, 4–6.

CHAN HSL, HADDAD G, THORNER PS, DEBOER G, LIN YP, ONDRUSEK N, YEGER H AND LING V. (1991). P-glycoprotein expression as a predictor of the outcome of therapy for neuroblastoma. *N. Engl. J. Med*., **325**, 1608–1614.

CHIN K-V, UEDA K, PASTAN I AND GOTTESMAN MM. (1992). Modulation of activity of the promoter of the human MDR1 gene by Ras and p53. *Science*, **255**, 459–462.

CROOK T, WREDE D, TIDY JA, MASON WP, EVANS DJ AND VOUSDEN KH. (1992). Clonal p53 mutation in primary cervical cancer: assocation with human-papillomavirus-negative tumours. *Lancet*, **339**, 1070–1073.

CROOP JM, RAYMOND M, WABER D, DEVAULT A, ARCECI RJ, GROSP P AND HOUSMAN DE. (1989). The three mouse multidrug resistance (mdr) genes are expressed in a tissue-specific manner in normal mouse tissues. *Mol. Cell Biol*., **9**, 1346–1350.

FAKHARZADEH SS, TRUSKO SP AND GEORGE DL. (1991). Tumorigenic potential associated with enhanced expression of a gene that is amplified in a mouse tumour cell line. *EMBO J*., **10**, 1565–1569.

FINLAY CA. (1993). The mdm-2 oncogene can overcome wild-type p53 suppression of transformed cell growth. *Mol. Cell Biol*., **13**, 301–306.

FOJO AT, UEDA K, SLAMON DJ, POPLACK DG, GOTTESMAN MM AND PASTAN I. (1987). Expression of a multidrug-resistance gene in human tumors and tissues. *Proc. Natl Acad. Sci. USA*, **84**, 265–269.

GAVRIELI Y, SHERMAN Y AND BEN-SASSON SA. (1992). Identification of programmed cell death *in situ* via specific labelling of nuclear DNA fragmentation. *J. Cell Biol*., **119**, 493–501.

GINSBERG D, OREN M, YANIV M AND PIETTE J. (1990). Protein-binding elements in the promoter region of the mouse p53 gene. *Oncogene*, **5**, 1285–1290.

GOLDSTEIN LJ AND OZOLS RF. (1991). Blocking P-glycoprotein action. Contemporary Oncol. May/June, 38–47.

GOLDSTEIN LJ, GALSKI H, FOJO A, WILLINGHAM M, LAI S-L, GAZDAR A, PIRKER R, GREEN A, CRIST W, BRODEUR GM, LIEBER M, COSSMAN J, GOTTESMAN MM AND PASTAN I. (1989). Expression of multidrug resistance gene in human cancers. *J. Natl Cancer Inst*., **81**, 116–124.

GROS P, BEN-NERIAH Y, CROOP JM AND HOUSMAN DE. (1986). Isolation and expression of a complementary DNA that confers multidrug resistance. *Nature*, **323**, 728–731.

HARRIS CC AND HOLLSTEIN M. (1993). Clinical implications of the p53 tumor-suppressor gene. *N. Engl. J. Med*., **329**, 1318–1327.

HINDS PW, FINLAY CA, QUARTIN RS, BAKER SJ, FEARON ER, VOGELSTEIN B AND LEVIN AJ. (1990). Mutant p53 cDNAs from human colorectal carcinomas can cooperate with *ras* in transformation of primary rat cells: a comparison of the 'hot spot' mutant phenotype. *Cell Growth Different*., **1**, 571–580.

HOLLSTEIN M, SIDRANSKY D, VOGELSTEIN B AND HARRIS CC. (1991). p53 mutations in human cancers. *Science*, **253**, 49–53.

JULIANO RL AND LING V. (1976). A surface glycoprotein modulating drug permeability in chinese hamster ovary cell mutants. *Biochim. Biophys. Acta*, **455**, 152–162.

KONDO S, YIN D, MORIMURA T, ODA Y, KIKUCHI H AND TAKEUCHI J. (1994). Transfection with a bcl-2 expression vector protects transplanted bone marrow from chemotherapy-induced myelosuppression. *Cancer Res*., **54**, 2928–2933.

KONDO S, BARNETT GH, HARA H, MORIMURA T AND TAKEUCHI J. (1995). MDM2 protein confers the resistance of a human glioblastoma cell line to cisplatin-induced apoptosis. *Oncogene*, **10**, 2001–2006.

LEVINE AJ, MOMAND J AND FINLAY CA. (1991). The p53 tumour suppressor gene. *Nature*, **351**, 453–456.

LOWE SW, RULEY HE, JACKS T AND HOUSMAN DE. (1993). p53-dependent apoptosis modulates the cytotoxicity of anticancer agents. *Cell*, **74**, 957–967.

MA DD, DAVEY RA, HARMAN DH, ISBISTER JP, SCURR RD, MACKERTICH SM, DOWDEN G AND BELL DR. (1987). Detection of a multidrug resistant phenotype in acute non-lymphoblastic leukemia. *Lancet*, **1**, 135–137.

MOMAND J, ZAMBETTI GP, OLSON DC, GEORGE D AND LEVINE AJ. (1992). The mdm-2 oncogene product forms a complex with the p53 protein and inhibits p53-mediated transactivation. *Cell*, **69**, 1237-1245.

OLINER JD, KINZLER KW, MELTZER PS, GEORGE DL AND VOGELSTEIN B. (1992). Amplification of a gene encoding a p53-associated protein in human sarcomas. *Nature*, **358**, 80-83.

OLINER JD PIETENPOL JA, THIAGALINGAM S, GYURIS J, KINZLER KW AND VOGELSTEIN B. (1993). Oncoprotein MDM2 conceals the activation domain of tumor suppressor p53. *Nature*, **362**, 857-860.

OLSON DC, MARECHAL V, MOMAND J, CHEN J, ROMOCKI C AND LEVINE AJ. (1993). Identification and characterization of multiple mdm-2 proteins and mdm-2-p53 protein complexes. *Oncogene*, **8**, 2353-2360.

ONISHI Y, AZUMA Y, SATO Y,. MIZUNO Y, TADAKUMA T AND KIZAKI H. (1993). Topoisomerase inhibitors induce apoptosis in thymocytes. *Biochim. Biophys. Acta*, **1175**, 147-154.

REIFENBERGER G, LIU L, ICHIMURA K, SCHMIDT EE AND COLLINS VP. (1993). Amplification and overexpression of the MDM2 gene in a subset of human malignant gliomas without p53 mutations. *Cancer Res.*, **53**, 2736-2739.

RONINSON IB, ABELSON HT, HOUSMAN DE, HOWELL N AND VARSHAVSKY A. (1984). Amplification of specific DNA sequences correlates with multidrug-resistance in chinese hamster cells. *Nature*, **309**, 626-628.

SAFA AR, GLOVER CJ, MEYERS MB, BIEDLER JL AND FELSTAD RL. (1986). Vinblastine photoaffinity labelling of a high molecular weight surface membrane glycoprotein specific for multidrug-resistant cells. *J. Biol. Chem.*, **261**, 6137-6140.

SARKADI B, PRICE EM, BOUCHER RC, GERMANN V AND SCABOROUGH GA. (1992). Expression of the human multidrug resistance cDNA in insect cells generates a high activity drug-stimulated membrane ATPase. *J. Biol. Chem.*, **267**, 4854-4858.

SCHEFFNER M, WERNESS BA, HUIBREGSTE JM, LEVINE AJ AND HOWLEY PM. (1990). The E6 oncoprotein encoded by human papillomavirus types 16 and 18 promotes the degradation of p53. *Cell*, **63**, 1129-1136.

SCHURR E, RAYMOND M, BELL J AND GROS P. (1989). Characterization of the multidrug resistance protein expressed in cell clones stably transfected with the mouse mdrl cDNA. *Cancer Res.*, **49**, 2729-2734.

SIDRANSKY D, MIKKELSEN T, SCHWECHHEIMER K, ROSENBLUM ML, CAVANEE W AND VOGELSTEIN B. (1992). Clonal expansion of p53 mutant cells is associated with brain tumour progression. *Nature*, **355**, 846-847.

THIEBAUT T, TSURURO T, HAMADA H, GOTTESMAN MM, PASTAN I AND WILLINGHAM MC. (1987). Cellular localization of the multidrug-resistance gene product P-glycoprotein in normal human tissues. *Proc. Natl Acad. Sci. USA*, **84**, 7735-7738.

UEDA K, CARDARELLI C, GOTTESMAN MM AND PASTAN I. (1987). Expression of a full-length cDNA for the human 'MDRI' gene confers resistance to colchicine, doxorubicine, and vinblastine. *Proc. Natl Acad. Sci. USA*, **84**, 3004-3008.

VOGELSTEIN B AND KINZLER KW. (1992). p53 function and dysfunction. *Cell*, **70**, 523-526.

YIN D, KONDO S, TAKEUCHI J AND MORIMURA T. (1994). Induction of apoptosis in murine ACTH-secreting pituitary adenoma cells by bromocriptine. *FEBS Lett.*, **339**, 73-75.

ZASTAWNY RL, SALVINO R, CHEN J, BENCHIMOL S AND LING V. (1993). The core promoter region of the P-glycoprotein gene is sufficient to confer differential responsiveness to wild-type and mutant p53. *Oncogene*, **8**, 1529-1535.